# Case report: A case of Savolitinib in the treatment of *MET* amplification mutation advanced lung adenocarcinoma with rare bilateral breast metastasis

**DOI:** 10.3389/fonc.2024.1450855

**Published:** 2024-08-13

**Authors:** Rui Deng, Yan-ying Li, Liang-liang Bai, Li Zhou, Yong-Sheng Wang

**Affiliations:** ^1^ Division of Thoracic Tumor Multimodality Treatment, Cancer Center, West China Hospital, Sichuan University, Chengdu, Sichuan, China; ^2^ Department of Radiotherapy Physics & Technology, West China Hospital, Sichuan University, Chengdu, China

**Keywords:** case report, advanced lung adenocarcinoma, MET mutation, breast metastasis, Savolitinib

## Abstract

**Background:**

The distant metastasis of lung cancer primarily occurs in the bones, liver, brain, and lungs, while the breast is an extremely rare site of metastasis. There is very limited literature on the occurrence of breast metastasis from lung cancer, and metastatic lesions in the breast are prone to being misdiagnosed as primary breast cancer, requiring careful attention and differentiation in the clinical diagnostic and treatment process.

**Case summary:**

The patient, a 63-year-old female, initially presented with an *EGFR* exon 21 L858R mutated left lung adenocarcinoma in 2017, treated successfully with surgical resection and subsequent monitoring. The relapse of disease occurred in January 2020. Despite maintaining a prolonged progression-free survival (PFS) with first-generation EGFR-TKI Afatinib, disease progression occurred in 2022 without detectable resistance mutations. Transition to second-generation TKI Furmonertinib resulted in poor control, with rapid progression including unusual bilateral breast metastases that exhibited inflammatory breast cancer-like peau d’orange changes. Standard chemotherapy achieved only short-term stability. Upon detecting a *MET* amplification mutation, treatment with Savolitinib was initiated. Remarkably, this led to significant clinical and radiographic improvement, notably resolving the peau d’orange appearance and reducing multiple lesions across the body.

**Conclusion:**

This case underscores the importance of continuous genetic profiling and tailored treatment approaches in managing advanced lung adenocarcinoma, particularly when presenting with rare metastatic sites and complex genetic landscapes. The successful application of Savolitinib following the identification of a *MET* amplification mutation highlights its potential in overcoming resistance mechanisms in NSCLC, providing a significant therapeutic option for similarly challenging cases.

## Introduction

Lung cancer is the most common malignant tumor worldwide and one of the deadliest cancers (1). Among them, non-small cell lung cancer (NSCLC) accounts for more than 85% of lung cancer cases, with lung adenocarcinoma being the most common subtype (2). Distant metastasis of lung cancer mainly occurs in the bones, liver, brain, and lungs, while the breast is an extremely rare site for metastasis (3). Breast tissue is considered an unconventional site for lung cancer metastasis, which may be related to the rich lymphatic system and capillary network in the breast. Its physiological characteristics may influence the tendency of lung cancer metastasis (4). Existing literature reports very few cases of breast metastasis in lung cancer.

Savolitinib is a highly selective mesenchymal-epithelial transition factor (MET) tyrosine kinase inhibitor (TKI) used primarily for treating NSCLC with *MET* exon 14 skipping mutations (5). It operates by blocking the *MET* signaling pathway, which is crucial for tumor growth and metastasis. Clinical studies have shown that Savolitinib, particularly in combination with other epidermal growth factor receptor (EGFR) inhibitors, can overcome resistance mechanisms in NSCLC that have failed previous treatments (6).

This reports a case of advanced lung adenocarcinoma with multiple metastases, including metastasis to the breast, with *EGFR* and *MET* mutation. We discuss this rare case in conjunction with existing relevant literature, aiming to enhance understanding of such phenomena and provide support for clinical decision-making. Additionally, through the report and review of this rare case, we hope to uncover more characteristics of lung cancer metastasis to the breast, providing further clues for future clinical decision-making and research.

## Case presentation

The patient, a 63-year-old elderly female, was identified with an occupying lesion in the left lung in January 2017, which was subsequently excised. The postoperative pathology revealed lung invasive adenocarcinoma. Genetic testing of the postoperative tissue showed an *EGFR* exon 21 L858R mutation. As the patient’s disease was staged at IA (according to AJCC 8^th^ edition criteria) (7), postoperative targeted therapy was not administered. From July 2017 to January 2020, regular follow-up examinations showed no recurrence of the disease. In January 2020, a new solid nodule was detected in the left lung during a follow-up, suggesting recurrence, and the patient began treatment with afatinib. From January 2020 to October 2022, during the treatment period, regular examinations showed no disease progression.

**Figure 1:**
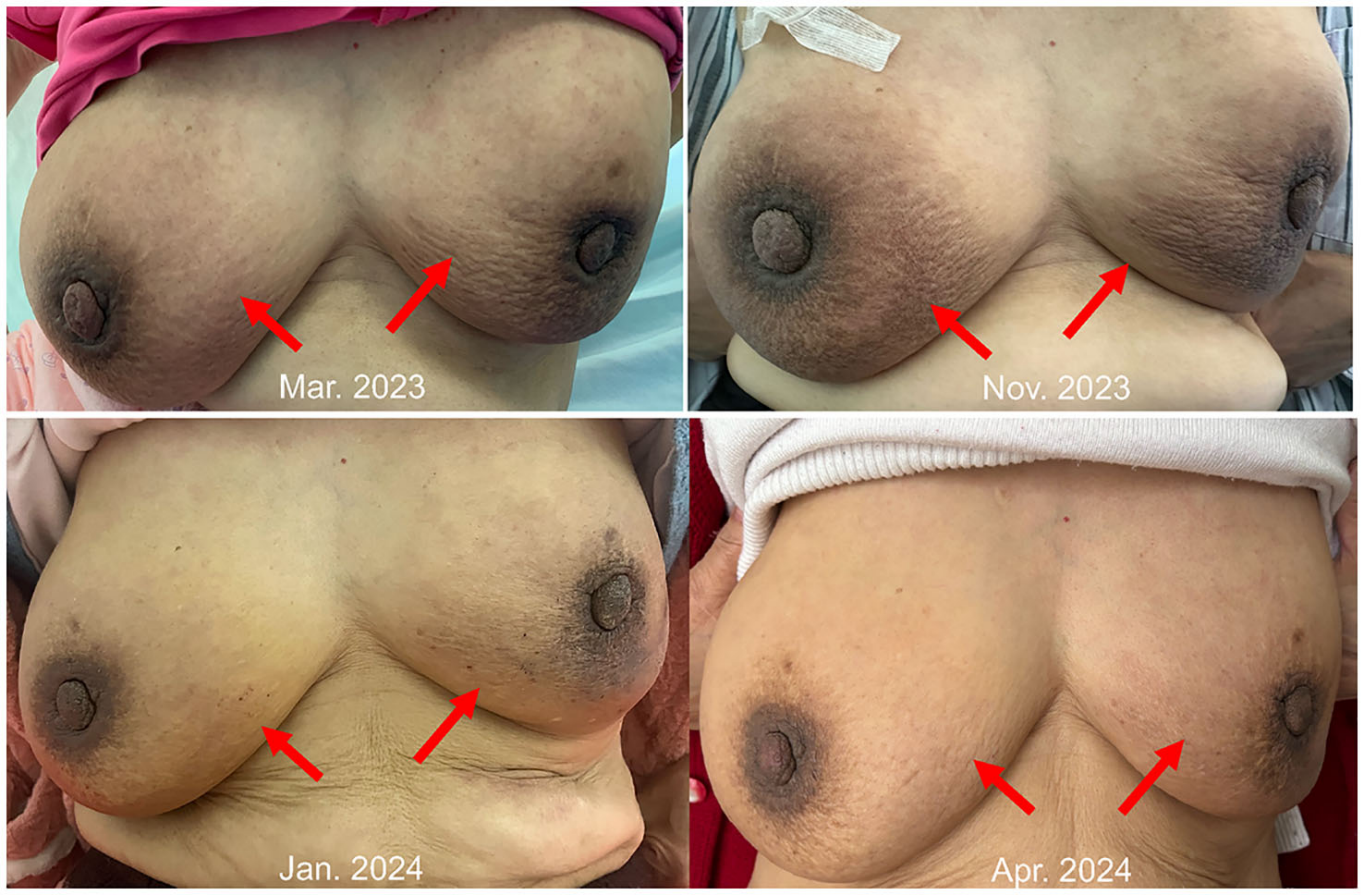
Peau d’orange appearance changes of bilateral breasts.

**Figure 2:**
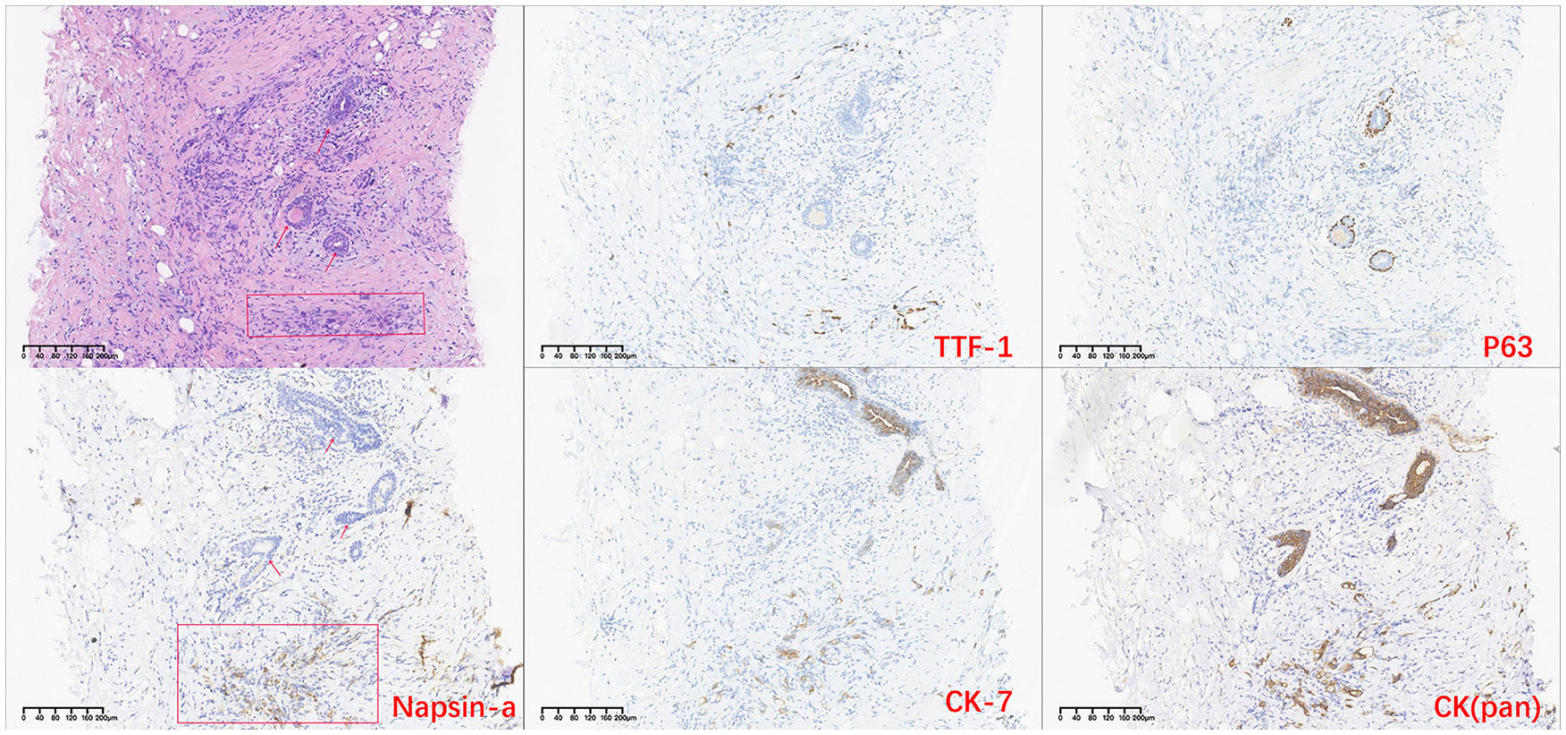
H-E stained histopathological and immunohistochemistry images of breast tissue. (The arrow indicates breast duct epithelium, and the rectangular box indicates tumor cells site).

However, in October 2022, multiple new lesions were found in both lungs during a check-up, leading to a determination of disease progression (PD) according to Response Evaluation Criteria in Solid Tumors (RECIST) criteria. Re-testing still showed the *EGFR* exon 21 L858R mutation without new resistance mutations. Considering the ineffectiveness of afatinib treatment, the patient was switched to Furmonertinib for targeted therapy. In January 2023, the patient experienced discomfort in the breasts, but visual examination of the skin showed no abnormalities. Breast ultrasound indicated ductal ectasia with Breast Imaging-Reporting and Data System (BI-RADS) category 4A, accompanied by multiple abnormal enlarged axillary lymph nodes. Further imaging studies in February 2023 revealed additional lymph node metastases in the cervical, axillary, mediastinal, and retroperitoneal regions, as well as breast and bone metastases. The therapeutic effect of Furmonertinib was poor, and the patient’s condition progressed comprehensively. In March 2023, the patient’s bilateral breast skin began to show radiating streaks with pigmentation, presenting a peau d’orange appearance (**Figure 1**). Biopsy of the left breast confirmed poorly differentiated adenocarcinoma consistent with pulmonary origin (**Figure 2**). Due to the ineffectiveness of Furmonertinib, from March 2023 to October 2023, the patient underwent chemotherapy based on pemetrexed combined with bevacizumab. The overall therapeutic effect during chemotherapy was stable disease (SD).

**Figure 3:**
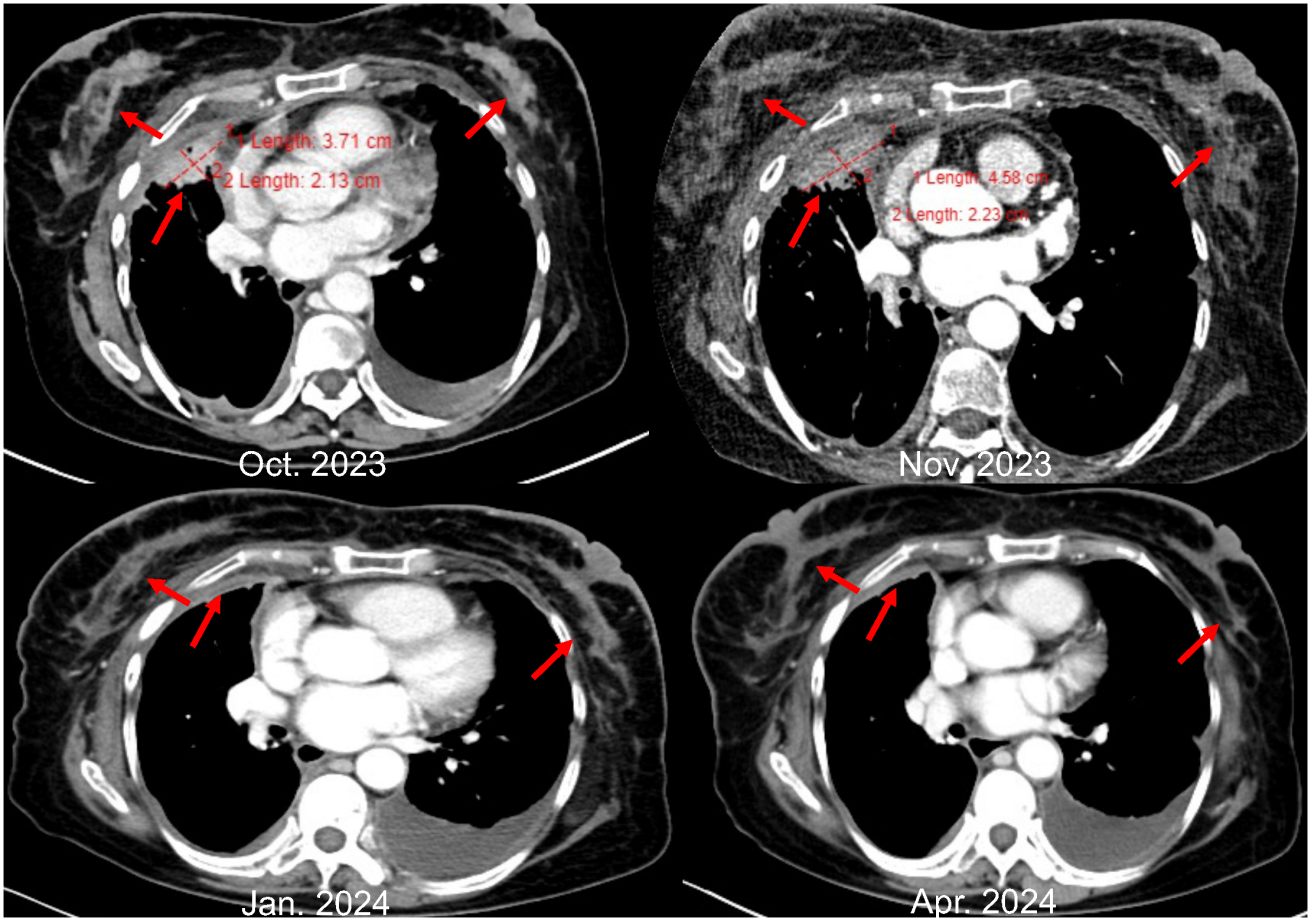
CT image changes of chest lesions.

**Figure 4:**
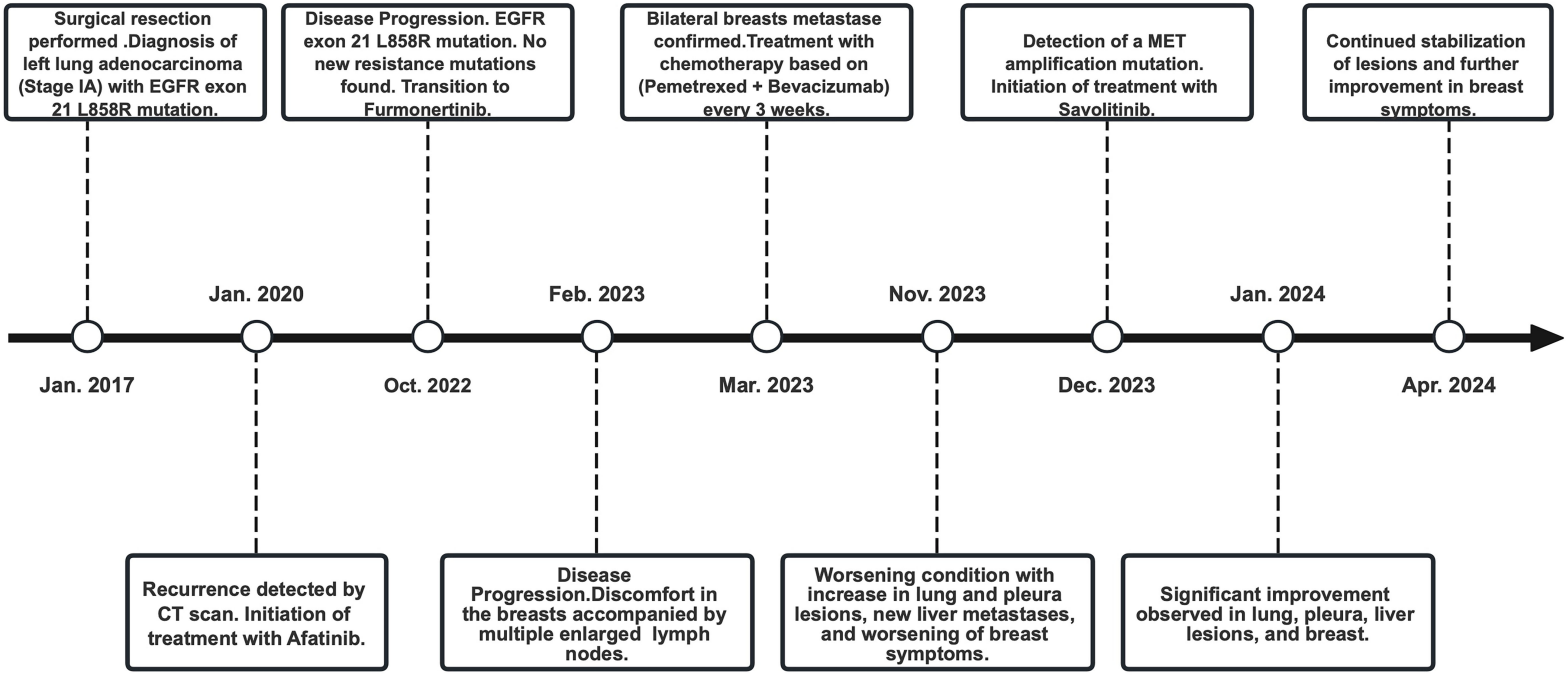
Timeline of the patient’s clinical course and therapeutic regimens.

Yet again, in November 2023, enhanced imaging examinations revealed an increase in the size of the lesions in both lungs and the pleura (**Figure 3**), new liver metastases, and worsening of the peau d’orange appearance of the breast, indicating further disease progression. Subsequent tissue biopsy and next-generation sequencing revealed, besides the EGFR L858R mutation, an additional *MET* amplification mutation. Consequently, from December 2023, the patient commenced treatment with Savolitinib. One month later, imaging showed significant reduction in the lesions in the lungs, pleura, and liver, achieving partial remission (PR), and a marked improvement in the peau d’orange changes of the breast. Three months later, imaging indicated stabilization of lesions throughout the body including pleura, with further improvement in the breast’s peau d’orange appearance. The timeline of this patient’s treatment journey was shown in **Figure 4**. As of the last update, the patient’s PFS with Savolitinib treatment has been maintained for five months.

## Discussion

A total of 29 cases of breast metastasis were reported in the 23 years from 2000 to 2023 (8). Although the metastasis of lung cancer to the breast is rare, when it occurs, it poses a series of challenges in terms of diagnosis and treatment. In many cases, this type of metastasis may be misdiagnosed as breast cancer, requiring clear pathological diagnosis and genetic analysis to guide the optimal treatment strategy (9). In certain instances, breast metastasis from lung cancer may exhibit a favorable response to standard treatment approaches for lung cancer (10). This further emphasizes the importance of understanding and identifying such rare metastases.

At first, Lung cancer cells can metastasize to distant sites through vascular and lymphatic systems. Due to the rich lymphatic and blood supply in the breast, it is plausible that lung cancer cells exploit these networks to establish metastatic sites (11). The anatomical and physiological characteristics of the breast, such as its extensive vascular network, may facilitate the lodging and growth of metastatic lung cancer cells.

Secondly, the tumor microenvironment, influenced by interactions between tumor cells and surrounding stroma, can promote metastasis. Factors secreted by tumor cells, such as VEGF (vascular endothelial growth factor) and TGF-β (transforming growth factor-beta), can modify the microenvironment to support tumor invasion and metastasis (12). The microenvironment of the breast might provide a fertile ground for lung cancer cells due to its unique stromal composition, which can be influenced by hormonal status.

Finally, the molecular drivers, which are probably the most applicable to this case. Despite significant advancements in the treatment of *EGFR*-mutated lung adenocarcinoma, such as targeted therapies for this mutation, its performance and mechanisms in breast metastasis remain unclear (13). Mutations in *EGFR* can activate downstream signaling pathways, such as PI3K/Akt and MAPK, enhancing the metastatic potential of tumor cells to colonize distant organs, including the breast (14). The *EGFR* mutation in lung adenocarcinoma patients may also influence the pattern of metastasis. By altering the interaction between tumor cells and the microenvironment, as well as affecting the invasiveness and migratory abilities of tumor cells, *EGFR* mutations can modify the metastatic patterns of lung adenocarcinoma (15). In certain case, a correlation has been explored between *EGFR* mutation and the metastasis of lung cancer to the breast (16). However, further research is still needed to determine the precise mechanisms underlying this relationship. The patient’s case, as highlighted, initially showed no additional genetic mutations when breast metastasis first appeared, suggesting that the breast lesions were primarily driven by the existing *EGFR* mutation. As the disease progressed, despite ongoing targeted therapy, a *MET* amplification mutation was eventually detected, indicating a shift in the driving force of the tumor’s biology.

*MET* amplification leads to overexpression of the *MET* protein, which in turn increases the activation of its tyrosine kinase domain. This activation triggers multiple downstream signaling cascades, notably the PI3K/Akt and MAPK pathways, which are crucial for cell survival, proliferation, and migration. These pathways are also involved in the epithelial-mesenchymal transition (EMT) (17, 18)​. *MET* and *EGFR* pathways exhibit significant crosstalk, which can contribute to compensatory signaling when one pathway is inhibited. When *EGFR*- tyrosine kinase inhibitor (TKI) therapies, such as erlotinib or gefitinib, are used to target *EGFR* mutations in NSCLC, cells with *MET* amplification can bypass the blocked *EGFR* signaling by activating alternative pathways through *MET*, leading to continued tumor growth and survival despite *EGFR* inhibition (19, 20). The crosstalk between *MET* and other receptor tyrosine kinases facilitates a bypass track for signaling, contributing to resistance against *EGFR*-TKIs. In clinical settings, patients initially responsive to *EGFR*-TKIs often develop resistance over time, which can frequently be traced back to secondary mutations or amplifications like *MET*. This resistance is characterized by a resurgence of proliferative and survival signaling despite ongoing *EGFR* blockade (21–23).

Besides resistance, *MET* amplification is directly linked to increased metastatic capabilities of tumor cells. *MET*-driven signaling enhances cell motility, invasion, and disruption of normal cellular adhesion, all of which are critical steps in the metastatic spread of cancer cells to distant organs (17, 18). Also, *MET* enhances the expression of vascular endothelial growth factor (*VEGF*), a key mediator of angiogenesis. The interaction between *MET* and *VEGF* signaling pathways boosts the vascularization of tumors, aiding their growth and the establishment of metastatic colonies (24). *MET*-driven signals can suppress immune surveillance mechanisms and modify the inflammatory response, creating a tumor-permissive environment that supports cancer cell survival and dissemination. This modulation of the tumor microenvironment not only aids in primary tumor growth but also plays a crucial role in preparing distant sites for metastatic colonization​ (25).

Therefore, it is pertinent to highlight the clinical significance of continuous genetic profiling (26). The dynamic nature of tumor genetics, exemplified in this case by the evolution from an *EGFR* mutation to an additional *MET* amplification, underscores the necessity of repeated genetic assessments. The development of resistance to targeted therapies, as observed in this patient with the initial response to *EGFR*-TKIs followed by progression, is a significant challenge in the treatment of lung adenocarcinoma. The resistance result required alternative therapeutic strategies, such as the use of *MET* inhibitors in cases where *MET* amplification is identified (19).

Savolitinib inhibits the activity of *MET* kinase, effectively blocking the *MET* signaling pathways. It targets the ATP binding site of the *MET* protein, which is essential for its kinase activity, thereby preventing the phosphorylation and activation of *MET*. This inhibition leads to a disruption in the downstream signaling pathways, particularly the PI3K/Akt and MAPK pathways, which are involved in promoting cell proliferation and survival. By inhibiting these pathways, Savolitinib impedes tumor growth and metastasis (6, 27)​. Savolitinib has progressed through various phases of clinical trials, showing beneficial efficacy and an acceptable safety profile in treating NSCLC patients with acquired resistance due to *MET* amplification. These studies highlight its potential not only as a monotherapy but also in combination with other targeted therapies, providing a robust strategy for tackling complex resistance mechanisms in advanced NSCLC (28–31).

In addition, there is a serious lack of understanding regarding effective treatment options for lung cancer metastasis to the breast. Due to the rarity of breast metastasis, it is difficult to conduct large-scale clinical trials to test different treatment strategies (32, 33). Currently, most treatment decisions are based on individual case reports and small case series. Each case contributes valuable insights into the potential responses to therapies and outcomes, forming a basis for future research and clinical guidelines. This is also one of the reasons why we report such rare case in this article, as we hope to provide more information for future understanding and treatment of similar cases.

## Conclusions

In summary, this case report is a valuable addition to the existing literature, particularly in understanding the potential of Savolitinib in treating NSCLC with *MET* amplification and managing rare metastatic presentations like bilateral breast metastasis. It highlights the importance of genetic profiling in guiding treatment decisions and the potential for new therapies to improve outcomes in challenging cases of lung adenocarcinoma.

## Data Availability

The raw data supporting the conclusions of this article will be made available by the authors, without undue reservation.
